# Superior vena cava blood flow and Doppler indices of brain sparing in late onset fetal growth restriction

**DOI:** 10.1038/s41598-025-94621-x

**Published:** 2025-03-27

**Authors:** Maria Stefopoulou, Jonas Johnson, Peter Lindgren, Ganesh Acharya

**Affiliations:** 1 Division of Obstetrics and Gynecology, Department of Clinical Science, Intervention and Technology (CLINTEC), Karolinska Institutet and Center for Fetal Medicine, Karolinska University Hospital, Stockholm, 141 86 Sweden; 2 Women’s Health and Perinatology Research Group, Department of Clinical Medicine, Faculty of Health Sciences, UiT-The Arctic University of Norway, Tromsø, Norway

**Keywords:** Blood flow, Doppler, Fetus, Fetal growth restriction, Hemodynamics, Superior vena cava, Biomarkers, Diseases, Medical research

## Abstract

Cerebral hemodynamic adaptation in fetal growth restriction (FGR) is primarily assessed using middle cerebral artery (MCA) Doppler and cerebroplacental (CPR) or umbilicocerebral ratio (UCR). The superior vena cava (SVC) blood flow may provide additional hemodynamic insights. Our objective was to evaluate fetal SVC blood flow velocities, pulsatility index for vein (PIV), volume blood flow (QSVC), and volume blood flow (Q)-based indices of fetal brain sparing in small-for-gestational-age (SGA) and FGR fetuses in the third trimester of pregnancy and compare with appropriately grown (AGA) fetuses. This was a prospective cohort study of 40 non-anomalous, singleton fetuses during 32 + 0 to 36 + 6 gestational weeks. Fetuses with abdominal circumference or estimated fetal weight below the 10th percentile were classified into SGA and FGR groups based on Delphi criteria. Doppler velocimetry of the umbilical artery (UA), umbilical vein (UV), fetal MCA and SVC was performed. UV and SVC diameters were measured, and their volume blood flows, i.e. QUV and QSVC were calculated. Both pulsatility index (PI)-based and Q-based indices of fetal brain sparing were calculated and compared to previously reported reference ranges for AGA fetuses using z-scores. In our study population, z-scores of SVC velocities (except the end-diastolic A-wave velocity) and PIV were significantly lower than the gestational age-specific mean values for AGA fetuses (p-values 0.005 to 0.018). Similarly, z-scores of SVC diameter (*p* < 0.001), QSVC normalized to fetal weight (QSVCw) (*p* < 0.001), blood flow volume-based QCPR (*p* < 0.001) were higher and QUCR (*p* < 0.001) was lower. However, z-scores of PI-based CPR (*p* = 0.195), UCR (*p* = 0.195), and the end-diastolic (A wave) velocity (*p* = 0.177) were not significantly different compared to AGA fetuses. Subgroup analysis demonstrated that the FGR fetuses (*n* = 21) had increased SVC diameter (*p* < 0.001), QSVCw (*p* < 0.001), QCPR (*p* < 0.001), UCR (*p* < 0.001), and decreased CPR (*p* < 0.001), QUCR (*p* < 0.001) and SVC PIV (*p* = 0.030), but no significant change in velocities was observed compared to AGA fetuses (*n* = 98) of similar gestational age. The SGA fetuses (*n* = 19) had decreased SVC S velocity (*p* = 0.013), D velocity (*p* = 0.005), TAMxV (*p* = 0.030), PIV (*p* = 0.005), QUCR (*p* = 0.014), and increased SVC diameter (*p* = 0.026), QSVCw (*p* = 0.034) and QCPR (*p* = 0.014) in comparison to AGA fetuses. When compared to SGA fetuses, the FGR fetuses had significantly lower QUVw (60.5 ± 19.7 vs. 80.1 ± 20.2 ml/min/kg, *p* = 0.004), QUCR (0.79 ± 0.45 vs. 1.34 ± 0.52 *p* < 0.001) and birthweight (2181 ± 577 vs. 2848 ± 330 g, *p* < 0.001) but higher QSVCw (91.82 ± 39.56 vs. 65.53 ± 17.79 ml/min/kg, *p* = 0.039) and QCPR (1.63 ± 0.74 vs. 0.90 ± 0.45, *p* < 0.001). In conclusion, third-trimester fetuses < 10th percentile had significantly increased SVC diameter, resulting in increased QSVCw in SGA and FGR despite reduced or unchanged TAMxV. Significantly altered QCPR and QUCR confirmed circulatory redistribution with increased brain and upper body venous return both in FGR and SGA fetuses. However, as the magnitude of increase in QSVCw and QCPR was significantly larger in FGR compared to SGA fetuses, it could be potentially used as a quantifiable marker to differentiate FGR from SGA. The role of SVC Doppler in refining the diagnosis of late FGR should be further investigated.

## Introduction

Doppler evaluation of placental and fetal blood flow is crucial for distinguishing the small-for-gestational-age (SGA) from growth-restricted fetuses (FGR)^1^. It helps interpret hemodynamic fetal adaptation to hypoxia, predict prognosis, and ensure appropriate clinical management of a compromised fetus^2,3^. Umbilical artery (UA) Doppler velocimetry is the cornerstone for assessing fetal well-being in early FGR^4–6^, but UA Doppler parameters are often normal in late-onset FGR^7^.

The middle cerebral artery (MCA) Doppler identifies the brain sparing effect^8^. The cerebroplacental ratio (CPR) or its reciprocal, umbilicocerebral ratio (UCR) has been used to evaluate this circulatory adaptation, especially during the third trimester of pregnancy^9–13^. SGA fetuses with brain sparing are at higher risk for adverse neurodevelopmental outcomes^14^. Cerebral blood flow redistribution is associated with adverse perinatal outcomes in late preterm FGR pregnancies^15^, and UCR (or CPR) has been suggested by some authorities to define a threshold for delivery in FGR after 32 gestational weeks^16^.

Fetal venous circulation adapts to hypoxia, with its evaluation providing essential clinical information. Studies demonstrated that assessing both arterial and venous circulations is crucial for evaluating fetal compromise, potentially altering the management of high-risk pregnancies^2^. Doppler velocimetry of ductus venosus (DV) is useful in monitoring and managing early FGR^17^, but not in late FGR.

Hemodynamic changes in late FGR are subtle compared to those observed in early FGR. It has been reported that decreased umbilical vein volume blood flow (QUV), which reflects placental blood flow, is more accurate in predicting adverse perinatal outcomes in late-onset FGR than CPR^18,19^. However, QUV is not routinely measured nor widely used in clinical practice.

Doppler ultrasound of superior vena cava (SVC) blood flow velocity waveforms has been previously shown to provide useful information regarding cardiac rhythm and function^20,21^. Changes in SVC blood flow, the venous connection between the brain and the heart, could reflect circulatory adaptations of fetuses with late-onset FGR. Our research group has established longitudinal reference ranges for fetal SVC Doppler indices and volume blood flow and introduced the concept of volume blood flow-based indices of fetal brain sparing in the second half of pregnancy^22,23^. We hypothesized that weight-indexed SVC volume blood flow (QSVCw) increases in late fetal growth restriction (FGR) and FGR fetuses have altered volume blood flow-based indices of brain sparing compared to appropriate for gestational age (AGA) fetuses.

The aims of the present study were to assess fetal SVC blood flow velocities, pulsatility index for vein (PIV), diameter, volume blood flow (QSVC), and volume blood flow-based indices of fetal brain sparing in SGA and FGR fetuses in the late third trimester of pregnancy and compare with AGA fetuses.

## Materials and methods

### Study population

This prospective cohort study was conducted at the Center for Fetal Medicine, Karolinska University Hospital, Stockholm, Sweden. Pregnant women were recruited from March 2023 until March 2024 as part of the ongoing international TRUFFLE 2 study (ISRCTN registry: 76 016 200.)^24^, investigating the optimal monitoring and timing of delivery in late-onset fetal growth restriction. The Swedish Ethical Review Authority approved the study (Ref. 2020–03298, approval date: 2020-10-27) with complementary ethical approval for SVC and UV blood flow measurements (Ref. 2022-01479-02, approval date: 2022-03-25). Pregnant women were consecutively recruited for fetal growth assessment after providing written informed consent. All methods, including ultrasound examinations, were performed according to relevant guidelines and regulations. Gestational age was established from first-trimester crown-rump length and confirmed during second-trimester ultrasound by measuring biparietal diameter.

Inclusion criteria included pregnant women ≥18 years of age with singleton pregnancies between 32 + 0 and 36 + 6 gestational weeks without chromosomal or major structural anomalies, fetal abdominal circumference (AC) or estimated fetal weight (EFW) below the 10th percentile for gestational age and normal short-term fetal heart rate variation (STV) on computerized cardiotocography (cCTG).

Exclusion criteria were absent or reversed UA end-diastolic blood flow, vaginal bleeding or leakage of amniotic fluid, any indication for immediate delivery required within 48 h, inability to understand Swedish or English, or lack of consent.

### Ultrasonography

Ultrasonography was performed using Voluson E10 and Expert 22 ultrasound system with a curvilinear abdominal transducer and frequency range of 2-5 MHz. After confirming fetal viability, fetal biometry was performed assessing EFW based on head circumference (HC), AC, and femur length (FL) using the Hadlock-3 formula^25^.

An experienced physician or midwife certified to perform ultrasound for the study obtained blood flow velocity waveforms from the UA and MCA as previously described^26,27^. Pulsed-wave Doppler velocity waveforms were recorded from each vessel in the absence of fetal movements, keeping the insonation angle of the Doppler close to zero and always below 30 degrees. At least three consecutive cardiac cycles were recorded for each blood vessel. The peak systolic velocity (S), end-diastolic velocity (D), and time-averaged maximum velocity (TAMxV) were measured, and the pulsatility index (PI) was calculated automatically by the software inbuilt in the ultrasound machine using the equation PI = (S-D)/TAMxV. The PIs of UA and MCA were used to calculate the CPR and UCR, expressed as the ratios MCA PI/UA PI and UA PI/MCA PI, respectively.

**Fig. 1:**
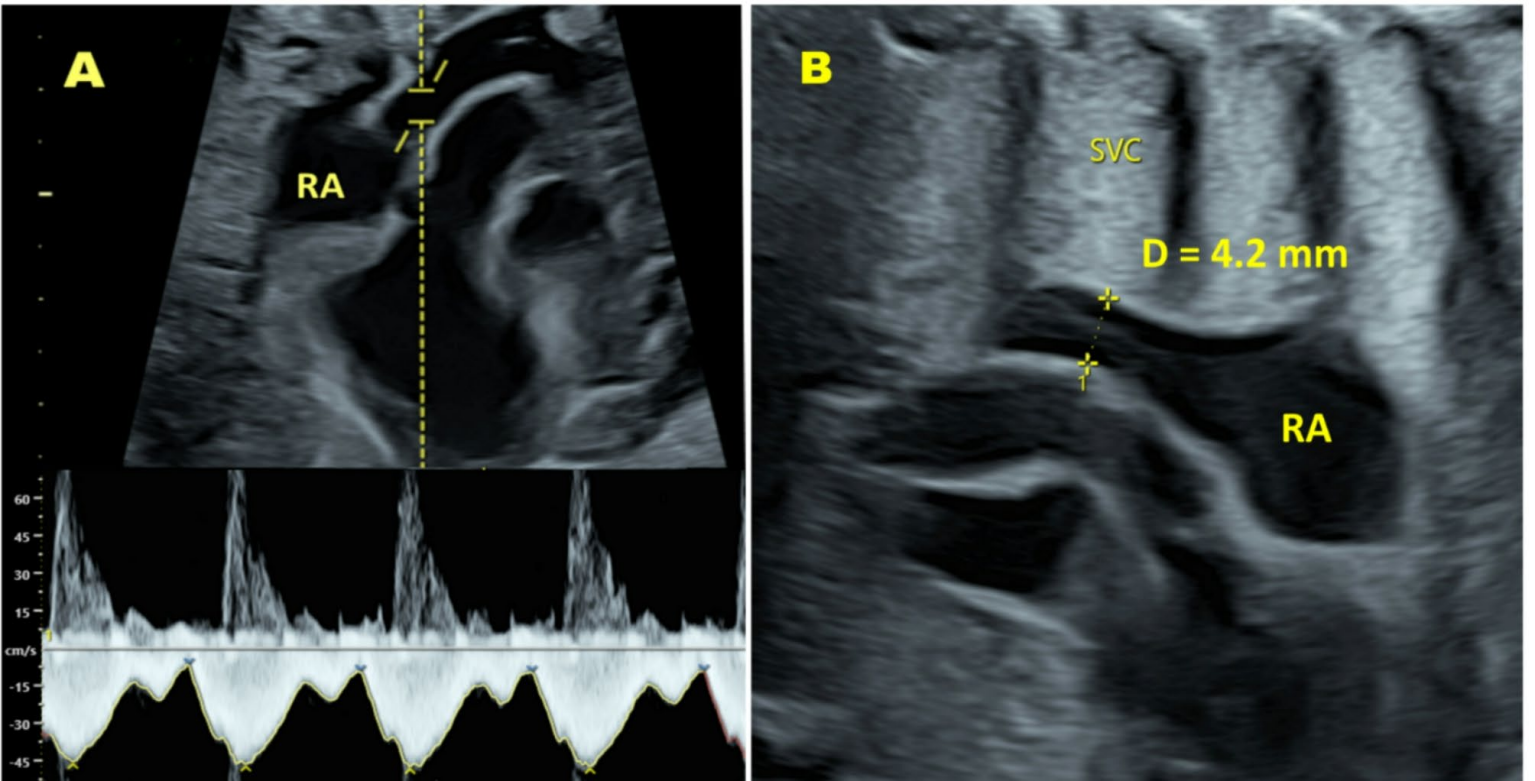
Ultrasound image demonstrating the technique of superior vena cava (SVC) blood flow velocities (A) and diameter (B) measurements. RA = Right atrium. D = SVC diameter. Maximum velocity envelope of the SVC Doppler velocity waveforms (below the baseline) is traced. Doppler velocity waveforms above the baseline represent the ascending aorta velocities.

On the venous side, SVC (Fig. 1A) and UV Doppler velocities were recorded, and SVC (Fig. 1B) and UV diameters were measured during the follow-up visit of the TRUFFLE 2 study by a single experienced operator (MS), as previously described^28,29^.

The UV Doppler velocity waveform was obtained at the umbilical cord free loop and TAMxV was measured for a period of 2–4 s. The SVC peak systolic (S) velocity, peak diastolic (D) velocity, TAMxV, and end-diastolic velocity during the atrial contraction (A) were recorded. The pulsatility index for vein (PIV) was calculated using the formula (S-A)/TAMxV as previously described^30^.

The SVC and UV inner diameters were measured in a perpendicular insonation using two-dimensional ultrasound. Both vessel side walls were well-defined and seen as hyperechogenic lines^28,29,31,32^. The UV diameter was measured at the umbilical cord segment where the UV Doppler velocity measurement was performed. The SVC diameter was measured in a long-axis view just before it enters the right atrium with simultaneous visualization of the ascending aorta. The caliper placement is shown in Fig. 1B. We performed SVC diameter measurements during the systolic phase of the cardiac cycle when the diameter is at its widest, and the vessel walls appear more well-defined.

Technically best measurements were accepted as reliable values for UV and SVC diameters.

UV volume blood flow (QUV) was calculated as: QUV (ml/min) = 0.5*UV TAMxV*UV CSA *60, as described previously^32^ and the SVC volume blood flow (QSVC), was calculated according to the formula: QSVC (ml/min) = 0.7*SVC TAMxV*SVC CSA*60, where CSA is the cross-sectional area of the vessel and is calculated as, CSA (cm^2^) = p*(vessel diameter/2)^2^, 0.5 and 0.7 represent the spatial blood velocity profiles of the respective vessels^28^. QSVCw was calculated as QSVC/EFW, QCPR as QSVC/QUV and QUCR as QUV/QSVC. Data on baseline characteristics of the study participants and pregnancy outcomes were obtained from the electronic medical records. Neonates with birthweight less than third percentile for the gestational age, based on the gender-specific reference charts constructed for the Scandinavian population, were considered growth-restricted^33^.

### Definition of subgroups of the study

Fetuses with EFW or AC between the 3rd and the 10th percentiles for the gestational age with normal UA Doppler and normal CPR and UCR were defined as SGA. FGR was defined according to the Delphi criteria for late FGR (≥ 32 weeks)^3^, i.e., AC (or EFW) < 3rd percentile or AC (or EFW) < 10th percentile based on Hadlock formula^25^ and CPR < 5th percentile or UA PI > 95th percentile, using Doppler reference ranges for UA and CPR published by Acharya et al.^26,27^.

### Statistical analysis

IBM SPSS Statistics for Windows, Version 28.0. (IBM Corp., Armonk, NY, USA) and MATLAB R2023b (MathWorks, Inc., Natick, MA, USA) were used for data analyses. The SVC blood flow velocities, diameter, PIV, QSVC, QSVCw, CPR, QCPR, UCR and QUCR, were plotted against gestational age-specific reference charts for low-risk pregnancies with corresponding 5th, 50th and 95th percentiles from our previous publications^22,23,28^.

Z-scores (difference between observed and expected values divided by the fitted SD corrected for gestational age) of fetal parameters were calculated to assess their deviation from the gestational age-specific mean of AGA fetuses.

**Fig. 2:**
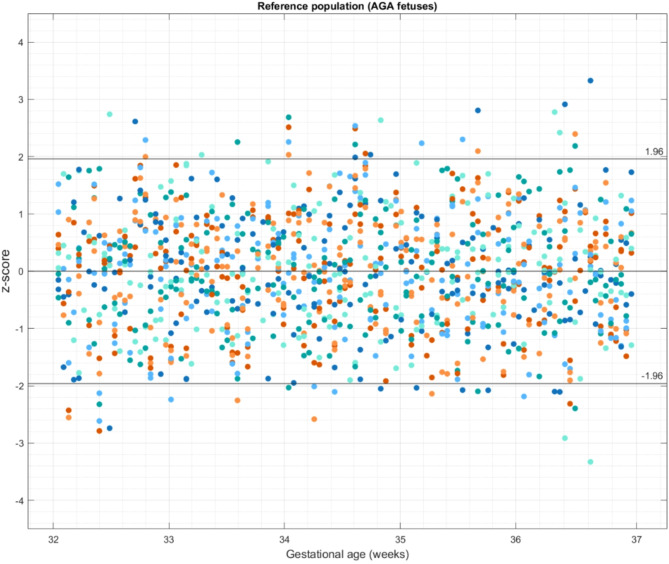
Scatter plot of z- scores of twelve fetal parameters obtained from appropriate for gestational age (AGA) fetuses demonstrating equal distribution above and below the zero line. The black lines represent standard deviation (SD) and each color of the dots represents a separate variable of superior vena cava (SVC) blood velocities, diameter, and volume blood flows.

The Z scores of the fetal parameters in AGA fetuses were equally scattered above and below the zero line throughout the gestational age range of 32 to 37 weeks. The number of z-scores falling out of ± 2 SDs was less than 5% of the measurements, as presented in Fig. 2. The mean of the Z-scores of the study population were compared with the reference population of AGA fetuses of similar gestational age using the nonparametric Mann-Whitney U test.

Receiver operating characteristic (ROC) analysis assessed the accuracy of antenatally measured QSVCw, QCPR and QUCR in SGA and FGR fetuses for predicting growth restriction at birth defined as newborn birthweight < 3rd percentile based on the gender-specific reference charts contructed for the Scandinavian population^33^. A p-value of < 0.05 was considered statistically significant.

## Results

A total of 45 women participated in this study. Five were excluded due to missing SVC Doppler or inadequate recording quality, leaving 40 for statistical analysis. Among them, 21 (52.5%) were FGR, and 19 (47.5%) were SGA.

Table 1 presents the baseline clinical characteristics of the study population and pregnancy outcomes. Among these women, 37 had first trimester ultrasound screening, one had noninvasive prenatal testing and one had chorion villous biopsy with normal results, whereas one woman opted not to have the first trimester screening. All of them subsequently had routine second trimester ultraound which did not show any fetal anomalies. One woman with preexisting type 2 diabetes mellitus, developed hypertension later during pregnancy. Three women developed preeclampsia, two gestational hypertension, and one gestational diabetes. The mean gestational age at delivery was 38 weeks, 16 (40%) neonates had a cesarean Sect. (9 elective and 7 emergency). Out of 24 vaginal deliveries, one was a vacuum extraction performed for fetal distress. The mean umbilical cord artery pH was 7.25, base excess was − 3.62 mmol/l and the respective mean values for the vein were 7.31 and − 3.32 mmol/l. Postnatally, 17 out of 40 newborns were under the third percentile for gender-specific Scandinavian reference charts^33^. Ten babies were admitted to the neonatal intensive care unit for observation and/or treatment; seven required continuous positive airway pressure (CPAP) for respiratory support, one needed nutritional support, one was treated for hypothermia, and one for infection. No perinatal mortality occurred. The average duration of hospital stay was 4.7 days (range 1 to 22 days).

**Table 1:** Baseline clinical characteristics of the study population of fetuses with estimated fetal weight below 10th percentile for gestational age (*n* = 40) and outcome data.

Variable	Median (IQR) (25th-75th), *n* %
Mother	
Maternal body mass index (kg/cm^2^)	22.7 (21.3–25.5)
Nullipara	22 (55%)
Systolic BP at inclusion	120 (110–131)
Diastolic BP at inclusion	79 (72–85)
Onset of labor	
Induction of labor	19 (47.5%)
Elective cesarean before onset of labor	9 (20.0%)
Spontaneous onset of labor	6 (0.15%)
Preterm premature rupture of membranes	1 (0.03%)
Mode of delivery	
Vaginal	24 (60.0%)
Cesarean section	16 (40.0%)
Neonate	
Gestational age at birth (weeks)	38.4 (36.5–39.8)
Birthweight (g)	2593 (2064–2987)
Length (cm)	46.0 (44.0–48.8)
Sex ratio (male/female)	19/21
Apgar score at 5 min	10 (10–10)

**Fig. 3:**
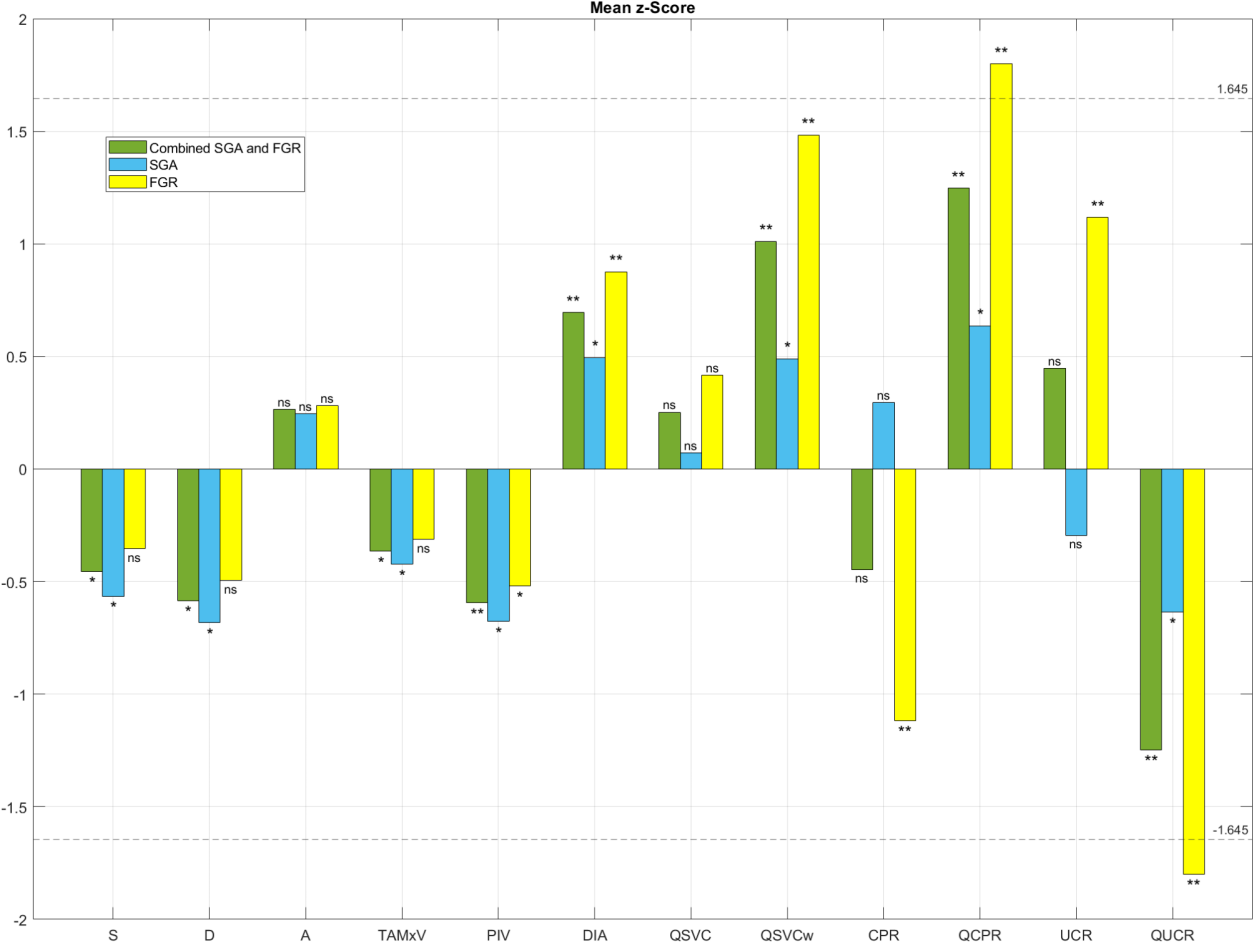
Bar chart demonstrating deviation of mean z-scores of superior vena cava blood velocities, diameter, volume blood flow and indices of fetal brain sparing in the total study population (i.e. combined SGA and FGR) of fetuses below 10th percentile for gestational age, small for gestational age (SGA), and growth restricted (FGR) fetuses compared to appropriate for gestational age (AGA) fetuses. Peak systolic velocity (S), diastolic velocity (D), time-averaged maximum velocity (TAMxV), end-diastolic velocity during atrial contraction (A), pulsatility index for vein (PIV), SVC diameter (DIA), volume blood flow (QSVC), volume blood flow normalized to fetal weight (QSVCw), cerebroplacental ratio (CPR), volume blood flow based cerebroplacental ratio (QCPR), umbilicocerebral ratio (UCR), volume blood flow based umbilicocerebral ratio (QUCR). NS = not significant, *p-value < 0.05 to 0.001, ** p-value < 0.001. Dashed lines represent 90% confidence intervals.

Z-scores of the total study population and the subgroups (FGR and SGA) compared to the reference population (AGA fetuses) are presented in Table 2, and as a bar chart in Fig. 3.

**Table 2:** Mean Z-scores of superior Vena Cava blood velocities, diameter, volume blood flow and indices of fetal brain sparing in the total study population of fetuses with estimated fetal weight below 10th percentile for gestational age, and subgroups of small for gestational age (SGA) and growth restricted fetuses (FGR) compared with appropriate for gestational age fetuses (*n* = 98) of similar gestational age.

Variable	Z-score total population (*n* = 40)	*p*-Value	Z-score SGA (*n* = 19)	*p*-Value	Z-score FGR (*n* = 21)	*p*-Value
S velocity	− 0.454	0.012	− 0.566	0.013	− 0.354	0.180
D velocity	− 0.584	0.005	− 0.683	0.005	− 0.495	0.122
A velocity	0.264	0.177	0.245	0.315	0.280	0.296
TAMxV	− 0.366	0.018	− 0.424	0.030	− 0.313	0.147
PIV	− 0.594	0.003	− 0.677	0.005	− 0.519	0.030
Diameter	0.695	< 0.001	0.495	0.026	0.876	< 0.001
QSVC	0.252	0.156	0.071	0.674	0.416	0.087
QSVCw	1.011	< 0.001	0.489	0.034	1.483	< 0.001
CPR	− 0.447	0.195	0.295	0.066	− 1.117	< 0.001
QCPR	1.246	< 0.001	0.635	0.014	1.799	< 0.001
UCR	0.447	0.195	− 0.295	0.066	1.117	< 0.001
QUCR	− 1.246	< 0.001	− 0.635	0.014	− 1.799	< 0.001

SVC = Superior vena cava, S Velocity = Peak systolic velocity, D Velocity = Peak diastolic velocity, A Velocity = End-diastolic Velocity during atrial contraction, TAMxV = A wave velocity during the atrial contraction, TAMxV = Time averaged maximum velocity, PIV = Pulsatility index for vein, QSVC = Superior vena cava volume blood flow, QSVCw = Weight-indexed superior vena cava volume blood flow, CPR = Cerebroplacental ratio, UCR = Umbilicocerebral ratio, QCPR = Volume blood flow-based cerebroplacental ratio, QUCR = Volume blood flow-based umbilicocerebral ratio.

**Fig. 4:**
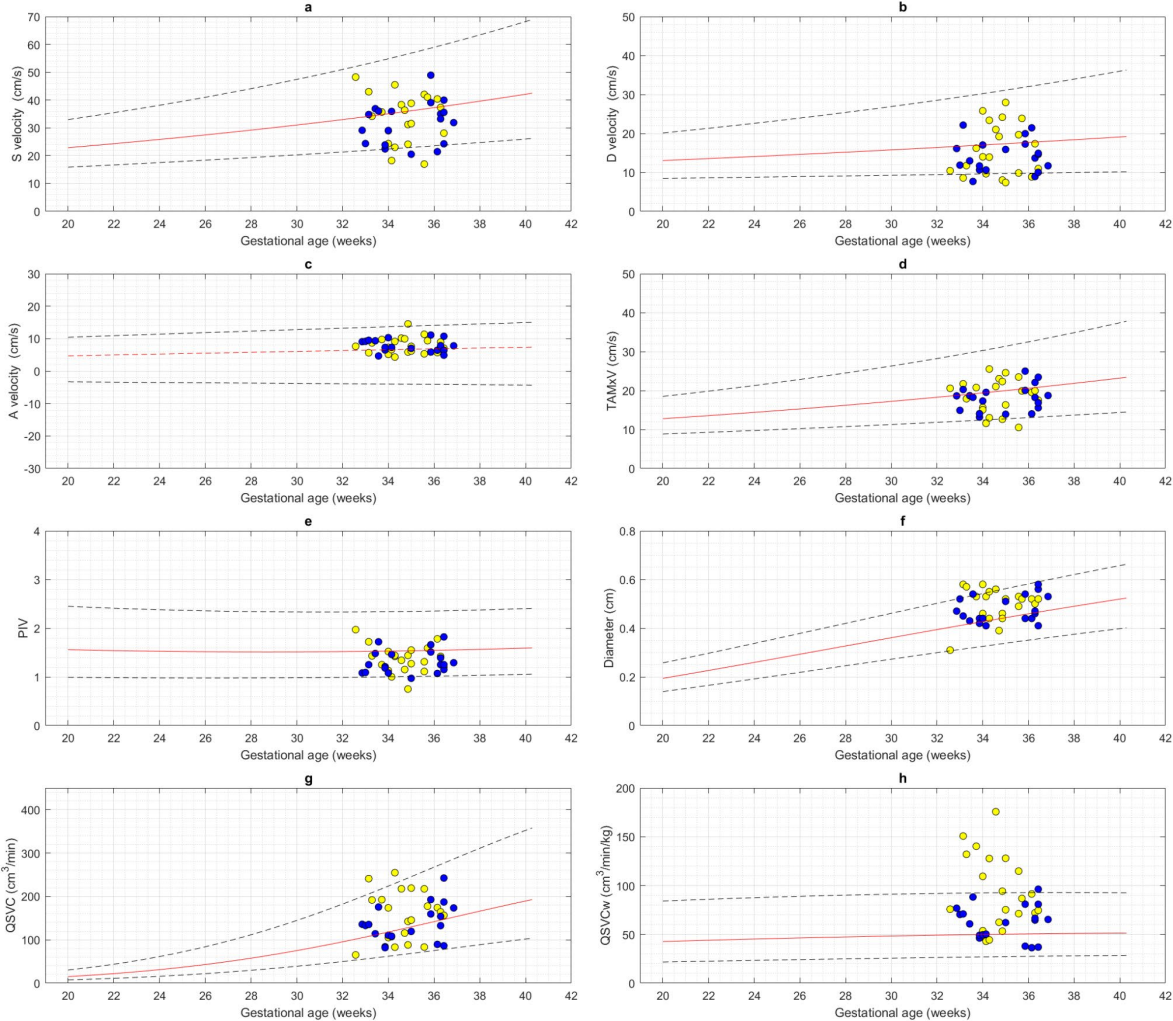
Superior vena cava blood velocities, diameter, and volume blood flows in small for gestational age (blue circles) and growth restricted (yellow circles) fetuses compared with appropriate for gestational age fetuses. The measurements of superior vena cava peak systolic (S) velocity, peak diastolic(D) velocity, time-averaged maximum velocity (TAMXV), and end-diastolic velocity during the atrial contraction (A velocity), pulsatility index for vein (PIV), diameter, volume blood flow (QSVC) and volume blood flow normalized to fetal weight (QSVCw) obtained from small for gestational age (SGA) and growth restricted (FGR) fetuses are plotted on gestational age specific reference charts for appropriate for gestational age fetuses. The red solid line represents the 50th percentile, the blue dashed lines represent the 5th and 95th percentiles.

**Fig. 5:**
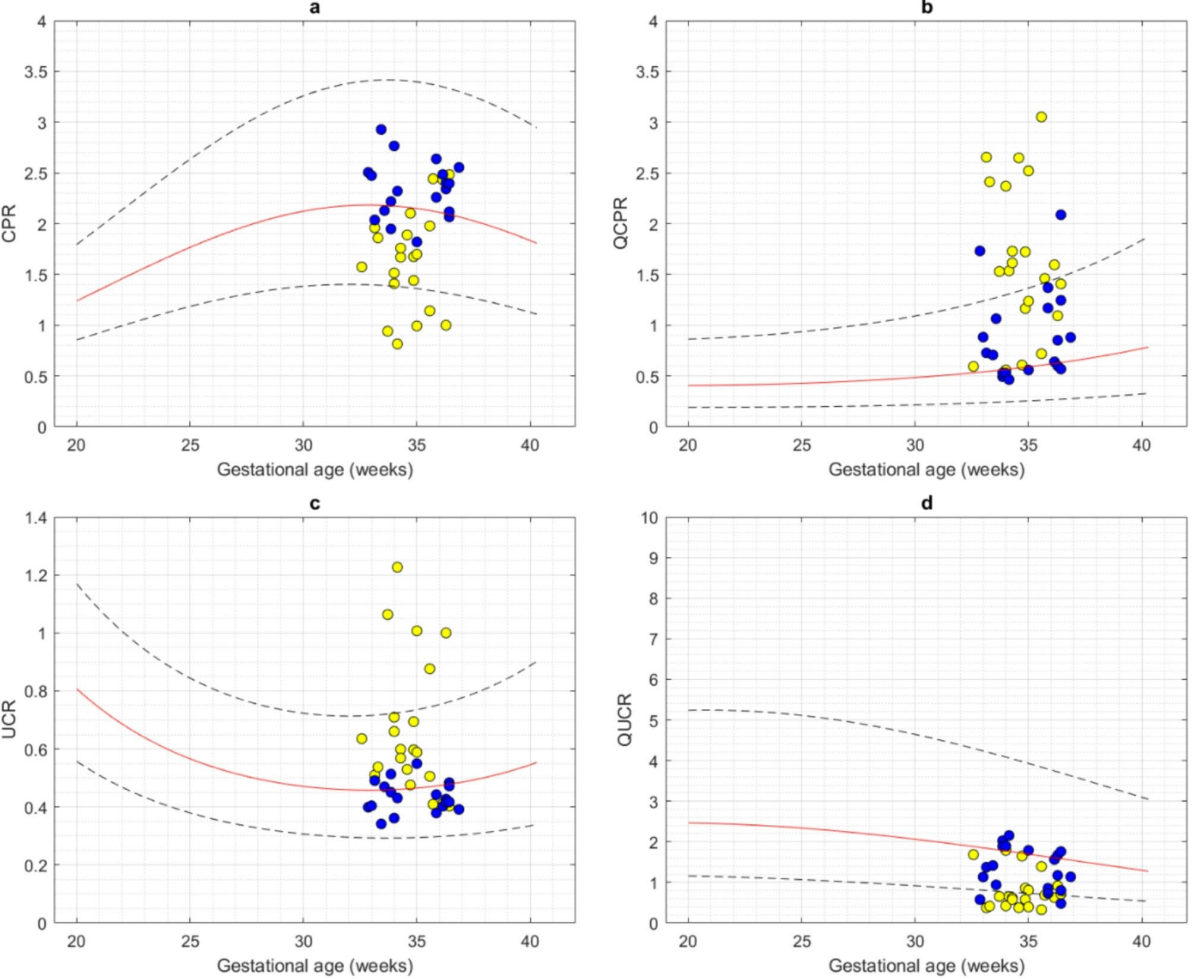
Indices of fetal brain sparing in small for gestational age (blue circles) and growth restricted (yellow circles) fetuses compared with appropriate for gestational age fetuses. The cross-sectional measurements of cerebroplacental ratio (CPR), cerebroplacental ratio based on volume of blood flow (QCPR), umbilicocerebral ratio (UCR) and umbilicocerebral ratio based on volume of blood flow (QUCR) obtained from small for gestational age (SGA) and growth restricted (FGR) fetuses are plotted on gestational age specific reference charts for appropriate for gestational age fetuses. The red solid line represents the 50th percentile, the blue lines represent the 5th and 95th percentiles.

Figures 4 and 5 display SVC velocities, PIV, diameter, QSVC, QSVCw, QCPR, QUCR, CPR, and UCR plotted on the previously constructed gestational age-specific reference charts.

When compared to SGA fetuses, the FGR fetuses had significantly lower QUVw (60.5 ± 19.7 vs. 80.1 ± 20.2 ml/min/kg, *p* = 0.004), QUCR (0.79 ± 0.45 vs. 1.34 ± 0.52 *p* < 0.001) and birthweight (2181 ± 577 vs. 2848 ± 330 g, *p* < 0.001) but higher QSVCw (91.82 ± 39.56 vs. 65.53 ± 17.79 ml/min/kg, *p* = 0.039) and QCPR (1.63 ± 0.74 vs. 0.90 ± 0.45, *p* < 0.001).

**Fig. 6:**
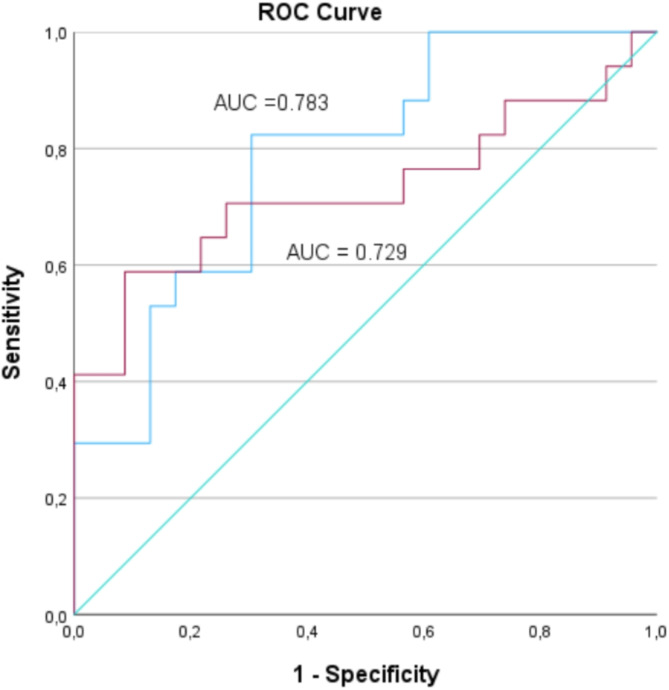
Receiver operating characteristic (ROC) curve for the prediction of fetal growth restriction at birth. QSVCW = Superior vena cava volume blood flow normalized by estimated fetal weight, QCPR = Volume blood flow based cerebroplacental ratio, QUCR = Volume blood flow based umbilicocerebral ratio, AUC = area under the curve. The red line represents the AUC of QSVCW and the blue line represents the AUC of QCPR (or QUCR).

The ROC analysis for the prediction of postnatally confirmed FGR demonstrated that the QSVCw had an area under the curve (AUC) of 0.729 (95% CI 0.554–0.904 *p* = 0.014) and volume blood flow-based indices of brain sparing (QCPR and QUCR) had an AUC of 0.783; (95% CI 0.641–0.924; *p* = 0.003) (Fig. 6).

## Discussion

Cerebral redistribution of fetal circulation is a well-studied adaptational mechanism in FGR^34^. The ratio between UA and MCA pulsatility indices is clinically used to assess the degree of brain sparing in fetuses with reduced intrauterine growth and stratify them into FGR and SGA groups, especially in the third-trimester^35^. However, systematic review of literature has shown that MCA Doppler has low accuracy in detecting neonatal acidosis^36^. We found that QSVCw increased significantly in SGA and in FGR fetuses. Altered volume blood flow-based indices calculated as a ratio of placental (UV) and upper body (SVC) venous return may provide more direct evidence of fetal brain sparing as the reference ranges have been recently established^23^. However, their clinical utility remains unexamined. This study showed that QCPR and QUCR are significantly altered both in FGR and SGA fetuses in the late third trimester,  while SVC blood velocities, except for the A velocity, differed significantly in SGA fetuses compared with AGA, but not in FGR fetuses.

Significantly increased QSVCw and reduced UV and pulmonary blood flow has been demonstrated in a study of 14 late FGR fetuses near term (mean gestational age 35.4 weeks; SD 4.2) using magnetic resonance imaging by Zhu et al.^37^. However, to our knowledge, the current study is the first to demonstrate increased QSVCw in late FGR using Doppler ultrasonography. In human fetuses, the QSVCw was increased in both SGA and FGR fetuses, but as the magnitude of change was significantly larger in FGR compared to SGA, it may represent a quantifiable marker of hemodynamic response to impaired fetal growth to differentiate FGR fetuses from SGA. Integrating SVC and UV blood flow measurements with MCA and UA Dopplers might refine the diagnosis of SGA/FGR subsequently improving perinatal outcomes.

Circulating blood volume, cardiac function, right atrial pressure, and ventricular compliance may affect venous return to the heart. However, the reduced or unchanged SVC TAMxV in SGA and FGR fetuses, indicates that the increased QSVCw results primarily from a larger SVC diameter. It is not surprising as cerebral vasodilation is a known mechanism of hypoxia-related increase in brain perfusion^34^. Increased right atrial pressure reduces the A wave velocity in precordial veins, such as in ductus venosus and inferior vena cava, due to the backward transmission of pressure waves during atrial contraction. However, we found slightly increased positive SVC A wave velocities and decreased SVC PIV in these late FGR fetuses, which may suggest that the atrial pressure was not increased. In contrast to our findings in late FGR fetuses, Fouron et al. have reported increased SVC systolic velocities and no significant increase in SVC diameter (0.36 ± 0.10 vs. 0.42 ± 0.06 cm) in 11 early FGR fetuses (mean gestational age 28.3 weeks, range 21 to 34 weeks) with absent UA end-diastolic flow compared to normal controls^38^. One possible explanation for this discrepancy might be more advanced hypoxia and severe placental insufficiency in their study population. Moreover, absent UA end-diastolic flow is very uncommon in late FGR, and none of the fetuses in our study had absent or reversed UA end-diastolic flow.

The significantly increased SVC diameter in FGR fetuses compared to AGA fetuses in our study is consistent with a recent study by Uzun Çilingir et al.^39^. Experimental studies have demonstrated increased shunting from SVC through foramen ovale to the left atrium, left ventricle, and subsequently to the upper body during hypoxia^40^. This could significantly increase the SVC diameter to accommodate increased venous return and, concomitantly, cause a decline in blood flow velocities in this highly compliant vessel.

The similar trends in SVC velocity changes but a more profound increase in QSVCw and QCPR (and decrease in QUCR) in FGR subgroup compared with SGA subgroup indicate that hemodynamic adaptations occur in a continuum and can be detectable even before the changes can be identified by MCA and UA Doppler. However, ROC analysis for the prediction of FGR, confirmed postnatally, demonstrated modest AUCs (< 0.80) for QSVCw as well as volume blood flow-based indices of brain sparing (QCPR and QUCR). Zhu et al. have reported a remarkably high AUC (0.94; 95% CI 0.87–1.0) for QSVCw using magnetic resonance imaging to measure SVC blood flow in late FGR fetuses^37^. However, their definition of growth-restricted newborn was different from ours and was based on any two of the following criteria: birthweight < 3rd percentile, ponderal index < 2.2 g/cubic centimeters and signs of placental under-perfusion on histology.

SVC Doppler velocimetry and blood flow measurement is feasible and shows clinical potential as a surrogate for brain perfusion in assessing fetal cerebral circulation. The evaluation of fetuses < 10th percentile for gestational age in the late third trimester demonstrated detectable changes in QSVCw, QCPR, and QUCR before abnormal CPR and UCR. This suggests that these Doppler measurements could be used as early markers of late-onset FGR. Most of the neonates in our study were delivered in stable condition and none of them had severe metabolic acidosis. Therefore, it could be argued that the currently used Delphi criteria^3^ to define late-onset FGR are not robust, although the stable neonatal condition at birth could also be related to our clinical practice of delivering cases of UA absent/reversed diastolic flow after 32 gestational weeks, and after 34 weeks if the UA pulsatility index exceeds the 95th percentile, according to our national guidelines. Further investigation is required to determine if the assessment of fetal SVC blood flow may help to distinguish which small fetuses are more likely at risk for adverse outcomes. Additional studies with sufficiently large population sample are needed to define the role of SVC Doppler in managing high-risk pregnancies, such as pregnancies complicated by diabetes, monochorionic twins, fetuses with intrauterine infection, severe anemia etc.

It is important to consider the strengths and limitations of our study. A single operator performed all the SVC and UV diameter measurements and Doppler velocimetry in this study eliminating the intra-observer variability. The velocity measurements were performed at a lowest possible angle of insonation. Although we accepted measurements with an insonation angle below 30 degrees (which might introduce an error of up to 13%), vast majority of the measurements had a lower angle of insonation (< 20 degrees) with a smaller (6%) risk of error, and angle correction was used. The same physician analyzed the SVC Doppler waveforms to establish longitudinal reference intervals of SVC blood velocities and PIV and volume blood flow in the previous studies^21,22,27^, which could be considered a strength of our study. However, as the ultrasound measurements are substantially operator-dependent, having a single operator might potentially limit the generalizability of our findings to clinical settings, where several clinicians are likely to perform ultrasound examinations. In addition, some selection bias can be expected in our cohort as women who could not speak and understand Swedish or English language were not included. Another limitation is that the measurement of SVC and UV blood flow was done at a single point in gestation rather than longitudinally, which does not allow serial assessment of fetal development and hemodynamics across gestation, potentially impacting the robustness of our observations. Therefore, our findings need replication and further validation before their wider clinical application.

The precision of volume blood flow measurement is always a limitation due to the squared vessel diameter in the mathematical equation used for calculating the cross-section area of blood vessel, which amplifies the possible minor measurement errors. However, SVC and UV are relatively large blood vessels, especially in the third trimester, and are easy to visualize and measure using modern ultrasound equipment. Moreover, SVC diameters were increased in SGA and FGR fetuses, which could further improve their measurement precision.

The sample size of our study was relatively small and a priori sample size calculation was not performed because of the unknown effect size due to lack of relevant studies published previously. A priori sample size calculation is not considered a prerequisite for an exploratory study. A post-hoc power calculation with CLINCALC software (https://clincalc.com/Stats/Power.aspx) using sample sizes of the study population (*n* = 40) and the reference population (*n* = 98) and the means and SD values of their weight-indexed fetal SVC blood flow (79.33 ± 33.12 ml/min/kg vs. 56.80 ± 25.12 ml/min/kg) and volume blood flow based index of brain sparing QCPR (1.28 ± 0.705 vs. 0.69 ± 0.366) from our study shows that it had a 90.2% and 99.2% power, respectively for QSVCw and QCPR, to detect the difference between the study population and the reference population with an alpha of 0.01.

## Conclusion

Third trimester fetuses < 10th percentile had significantly increased SVC diameter, which resulted in increased QSVCw in SGA and FGR fetuses despite slightly reduced or unchanged TAMxV. Significantly altered volume blood flow-based indices of brain sparing (QCPR and QUCR) confirmed redistribution of circulation with increased brain and upper body venous return both in FGR as well as SGA fetuses. However, as the magnitude of increase in QSVCw and QCPR was significantly larger in FGR fetuses compared to SGA, it could be potentially used as a quantifiable marker to differentiate FGR from SGA. The role of SVC Doppler in refining the diagnosis of late FGR should be further investigated.

## Data Availability

The datasets generated during and/or analysed during the current study are available from the corresponding author on reasonable request.
